# Atlanta Medical College

**Published:** 1867-06

**Authors:** 


### EDITORIAL AND MISCELLANEOUS

### ATLANTA MEDICAL COLLEGE.

Since the opening of the present Course of Lectures, the 6th instant, a material change, in the amount of teaching offered by the Institution, has been adopted by the Faculty.

At the conclusion of the present regular session, about the first of September next, a course of Practical or Clinical Instruction, with daily examinations and six lectures a week, on the several fundamental branches of Medicine, will be commenced, and will continue until the next regular course, commencing the first of May, 1868.

This additional labor will be assumed by the Faculty of the Institution.

FACULTY.

A. MEANS, M. D., Prof. of General and Medical Chemistry

D. C. O'KEEFE, M. D., Prof. of Principles and Practice of Medicine.

W. S. ARMSTRONG, M. D., Prof. of General and Special Anatomy.

H. V. M. MILLER, M. D., Prof. of Obstetrics and Diseases of Women and Children.

W. F. WESTMORELAND, M. D., Prof. of Principles and Practice of Surgery.

EBEN HILLYER, M. D., Prof. of Physiology.

J. G. WESTMORELAND, M. D., Prof. of Materia Medica and Therapeutics.

G. L. JONES, M. D., Demonstrator of Anatomy.

One Annual Commencement for conferring degrees will be held at the conclusion of each regular course of Lectures, about the first of September.

Fees for the Regular Course, from May 1st, to Sept. 1st, $105.
Matriculation, (required only once in the Institution) ....5.

Dissecting Ticket,..................................10.
Graduation Fee,.....................................25.
Fees for the Practical Course of 8 months,..............15.

In announcing to the medical profession and the public the above important modification of their course of instructions, the Faculty of the Medical College deem it proper to assign some of the reasons which have prompted its adoption.

The defects of the system of medical education generally pursued in the United States, have been long felt and deplored by the profession. Attempts to remedy them have been made at different times, by different medical schools, and valuable suggestions have been offered for improvement by individuals not connected in any way with the business of teaching: but from a variety of causes, these laudable attempts have failed; the useful and well meant suggestions have not been carried into practice; and, substantially, the same system which was adopted a century ago is still pursued; improved, it is true, by greater experience and proficiency, and enriched by more valuable appliances, but, it is believed, without radical change.

The imperfections of that system, becoming every year more apparent, consist mainly in the shortness of the time devoted to the acquisition of elementary principles, and the entire or partial want of opportunity to witness the practical application of them. A longer term of study and more extended and varied clinical observations are necessary to perfect the education of physicians, and to elevate the standing of the profession.

Under a different system of government, these objects might be secured by legal requirement; but in this country, where each State assumes to regulate, or to learn without regulations, the subject of education within its own limits, a general law, which alone would be useful, is impracticable. Medical Colleges are so numerous, and their interests so conflicting, that no one of them can successfully inaugurate the needed reformation: so that the only plan of securing greater perfections of medical education, which holds out reasonable hopes of success, is to offer the student increased facilities for the acquisition of knowledge; to place within his reach, at small cost, ample opportunities to learn, thoroughly, the theory and practice of every branch of his profession, and, by this appeal to honorable ambition and to his interest, induce him, by the longer prosecution of his studies, to avail himself to the utmost of these advantages.

The instruction conveyed in the customary series of lectures, in many of the existing Medical Colleges, is as full and complete as genius and learning can make it, in the brief period devoted to it; but such a map of facts, upon so many different subjects, is necessarily so crowded upon the attention of the student, as to leave him little time for the patient study of books, or practical anatomy, or for the more important investigation of disease as it actually occurs in the patient. It is proposed, in this institution, to retain all the advantages confessedly secured by the ordinary course of lectures of four months, devoting as much attention as that time will admit to practical anatomy; and utilizing, as far as possible, the extraordinary facilities for clinical instruction which this City affords. In addition to this curriculum, customary in all Medical Colleges, clinical lectures will be delivered daily, throughout the year, to all matriculants of the College; and daily recitations or examinations, covering the entire course of study expected of them, conducted by the members of the Faculty who teach during the regular term, the subjects to which they respectively relate.

It will be perceived, that this plan virtually extends the period of instruction to twelve months.

Attendance during the entire time will not be required as a condition of graduation. It is not proposed, in fact, at least for the present, to change the qualifications expected of candidates for the degree of Doctor of Medicine; but the advantages of the scheme are so great and so apparent, that it is not doubted that a large number of students who aspire to distinction, and determine to derive success in their profession, will voluntarily hasten to profit by them. The proposition *invites* to the pupil a long course of well directed study, encouraged and stimulated by daily examinations, by experienced teachers in the presence of his associates. Aided by the specimens and preparations in the College Museum, (which in a short time he has not time to examine) and by abundant material, he may select the most convenient season and the most leisure hours for the complete and satisfactory study of practical anatomy; while there will be, daily, submitted to his inspection, specimens of every variety of disease, in every stage of progress, confirming the lessons learned from his books, or which fall from the lips of his teachers.

The Faculty have been led to the adoption of this plan of teaching by a conviction of its necessity and usefulness, as well as by the remarkable facilities which the city affords for its successful prosecution. Indeed, they would be recreant to their duty if they neglect to mark the advantages subservient to the cause of science and medical education.

The population of Atlanta is now estimated at twenty-five thousand; it is growing with wonderful rapidity, and, in a few years, will be by far the largest city in the State, affording, certainly, equal opportunities with any other city of the same size for the cultivation of medical science. But from the peculiar circumstances of a large number of its inhabitants, it furnishes to the clinical lecture-room, and to the beds of its hospitals, more than the usual proportion of patients. From twenty to thirty of these present themselves every morning at the dispensary for examination and relief, furnishing illustrative examples, during the year, of almost every form of disease that the physician or surgeon will be likely to meet with in his future practice, and amounting in the aggregate to very nearly ten thousand prescriptions per annum.

Independent of the City Hospital, which is more remote, there is in the College grounds a hospital averaging between one and two hundred patients, the inmates of which are not all drawn from the city, but are sent here from many of the surrounding counties for treatment of the graver forms of surgical and other diseases, and women who resort thither for shelter and support during their confinement.

From these copious sources of supply, likely to become more abundant with each succeeding year, the Faculty will be able to illustrate the daily clinical lectures during the entire winter course. Indeed, it rarely happens that there are not found in the hospital, or attending the dispensary instruction, examples of every stage of venerial disease, of scrofula, of disease of the eye, of uterine disease, and many others, sufficient to justify a series of lectures on each of these specialities.

The number and variety of surgical cases submitted to the inspection of the pupil, and the number of surgical operations performed in his presence, is proportionably greater. The surgical clinic is in fact richer, if not more important, than the obstetrical or the medical, for the reason that the patients are drawn from a wider area, and are generally the subjects of graver injuries or of malignant diseases, demanding the highest operative skill for their relief or removal.

With these abundant means at their disposal, and with the determination most efficiently to employ them, the Faculty confidently invite the coöperation of the profession in the attempt to make Atlanta the chief center of medical education in the Southern States. The mildness of its climate, the salubrity of its atmostphere, the purity of its water, and the cheapness of living, are important and well known advantages, which, together with the superior opportunities now offered for the acquisition of professional knowledge, must commend it to the public as a most suitable place to *begin* to *prosecute* and to *complete* a medical education.

The attention of the younger members of the profession who may not have had time or opportunity during their hurried course to study disease at the bed side, is respectively and earnestly invited to the advantages presented by this prolonged clinical course, and by the profusion of material by which it will be enriched. Nowhere in America or in Europe, can a limited number of students find a better field or fairer opportunity to lay the foundation for further usefulness and honorable distinction.

To the graduates of this and other colleges, the usual courtesies are most cordially tendered.

---

We learn that the failure on the part of Dr. V. H. Taliaferro, of Columbus, Ga., to deliver the Annual Address at the last meeting of the Medical Association of Georgia, was caused by severe and protracted illness; and that his regrets at not being able to attend the meeting were much more profound than members' who desired to hear his address.

